# The Role of Gli1^+^ Mesenchymal Stem Cells in Osteogenesis of Craniofacial Bone

**DOI:** 10.3390/biom13091351

**Published:** 2023-09-05

**Authors:** Laidi Wu, Zhixin Liu, Li Xiao, Mi Ai, Yingguang Cao, Jing Mao, Ke Song

**Affiliations:** 1 Department of Stomatology, Tongji Hospital, Tongji Medical College, Huazhong University of Science and Technology, Wuhan 430030, China; 2 Department of Prosthodontics and Implantology, School of Stomatology, Tongji Medical College, Huazhong University of Science and Technology, Wuhan 430030, China; 3 Hubei Province Key Laboratory of Oral and Maxillofacial Development and Regen-Eration, Wuhan 430022, China

**Keywords:** Gli1, mesenchymal stem cells, osteogenesis, craniofacial bone

## Abstract

Glioma-associated oncogene homolog 1 (Gli1) is a transcriptional activator of hedgehog (Hh) signaling that regulates target gene expression and several cellular biological processes. Cell lineage tracing techniques have highlighted Gli1 as an ideal marker for mesenchymal stem cells (MSCs) in vivo. Gli1^+^ MSCs are critical for the osteogenesis of the craniofacial bone; however, the regulatory mechanism by which Gli1^+^ MSCs mediate the bone development and tissue regeneration of craniofacial bone has not been systematically outlined. This review comprehensively elucidates the specific roles of Gli1^+^ MSCs in craniofacial bone osteogenesis. In addition to governing craniofacial bone development, Gli1^+^ MSCs are associated with the tissue repair of craniofacial bone under pathological conditions. Gli1^+^ MSCs promote intramembranous and endochondral ossification of the craniofacial bones, and assist the osteogenesis of the craniofacial bone by improving angiopoiesis. This review summarizes the novel role of Gli1^+^ MSCs in bone development and tissue repair in craniofacial bones, which offers new insights into bone regeneration therapy.

## 1. Introduction

### 1.1. Characteristics of Gli1^+^ MSCs

Mesenchymal stem cells (MSCs) are conventionally considered as a heterogeneous population of cells that exist in various tissues. They are characterized by continuous self-renewal and multilineage differentiation and play a crucial role in maintaining tissue homeostasis [1,2]. MSC-mediated tissue regenerative therapy involves in vitro proliferation and transplantation of MSCs. However, these processes reduce cell viability, self-renewal ability, pluripotency, and genomic stability, thereby limiting the clinical application of MSCs in tissue regenerative therapy [3,4]. Endogenous stem cells facilitate tissue repair, making them a promising tool for tissue regenerative therapy. Therefore, it is necessary to establish a unique phenotypic fingerprint for MSCs for purification and labeling in vivo. This will allow the development of more effective regenerative therapies that harness the full potential of endogenous stem cells.

The Hedgehog (Hh) signaling pathway plays a significant role in embryonic bone development and bone remodeling throughout postnatal life by modulating MSC-mediated osteogenesis. Dysregulation of Hh signaling may result in various bone-related diseases, including osteoarthritis, osteoporosis, and bone defects [5]. Glioma-associated oncogene homolog 1 (Gli1), a transcription factor in the Hh signaling pathway, regulates the expression of Hh signaling target genes [6]. In line with the role of the Gli1 factors in Hh signaling, Gli1^−/−^ mutant mice seem to be normal, fertile, and can live past one year of age and show no obvious behavioral abnormalities [7,8,9]. Park et al. found that although Gli1^−/−^ mice had no gross abnormalities at birth, the survival rates during the first 10 days after birth were reduced. In addition, the growth rate and the body weights of the surviving Gli1^−/−^ mice were significantly lower than those of both the wild-type (WT) or Gli1^−/+^ mice [10]. Our previous study revealed that the overexpression of miR-342-3p in human umbilical MSCs could upregulate the expression of Gli1, alkaline phosphate (ALP), and osteocalcin (OCN). As a result of this upregulation, miR-342-3p can participate in osteogenesis by activating the TGF-β signaling pathway. Furthermore, miR-342-3p negatively regulates the suppressor of fused protein (Sufu), which has been identified as a suppressor of the Hh signaling pathway [11,12]. Transgenic mice are ideal models for amplifying Gli1 signaling and labeling Gli1^+^ MSCs. Through genetic labeling, researchers have confirmed that Gli1 can be used as a universal marker for MSCs from multiple tissues in vivo, and that Gli1^+^ MSCs are mainly distributed in the cardiopulmonary system, intestinal tract, liver, kidney, teeth, and bone [13,14,15,16,17,18]. Gli1^+^ MSCs also highly express typical MSC markers such as CD44, CD73, CD105, and SCA1, and exhibit colony-forming activity and multilineage differentiation ability [19,20,21]. We isolated Gli1^+^ cells from the outer membrane of the thoracic aorta of mT/mG/Gli1-CreERT2 mice. These cells are PDGFRα-, SCA1-, and CD34-positive, as well as capable of differentiating into adipocytes, osteoblasts, and myofibroblasts [18]. Using Gli1 as an in vivo marker, researchers can selectively target MSCs to investigate the functions of specific genes in the regulation of MSCs in vivo.

### 1.2. Mechnisms of Osteogenesis of Craniofacial Bone

Craniofacial bones have a unique embryonic origin and develop via two ossification patterns: intramembranous and endochondral. The craniofacial bones derived from the ectoderm consist of the frontal, parietal, occipital, maxillary, and parts of the mandibular bones, which depend on intramembrane ossification [22]. Craniofacial bones originating from the mesoderm include the mandibular condyle and skull base cartilages, which are formed via endochondral ossification [23]. Endochondral bones undergo differentiation of MSCs into cartilage to form the initial cartilage template, followed by vascularization and remodeling of the cartilage template to produce new bone tissue mineralization [24]. Bones display diverse formation patterns and signaling properties depending on their origin. Taking into account this heterogeneity in the investigation of bone biology and the development of clinical applications will facilitate the formulation of effective strategies for craniofacial bone repair.

Under pathological conditions, MSC-mediated osteogenesis is required for the regenerative repair of craniofacial bone defects. Craniofacial bones are susceptible to various conditions, such as trauma, infection, tumors, congenital diseases, and progressive deformable diseases. These conditions can significantly affect the function and appearance of the oral and maxillofacial systems, leading to a diminished quality of life for the affected individuals [25]. Bone tissue engineering is a promising approach that utilizes MSCs, growth factors, and bioactive carrier materials to induce new bone tissue formation [26]. MSCs facilitate tissue repair by differentiating into osteoblasts. Despite the obvious benefits associated with cell delivery for bone regeneration, the isolation, selection, and enrichment of MSCs, as well as the maintenance of the MSC phenotype in tissue culture dishes and the low survival rate after transplantation pose challenges for research [27].

In addition to the ossification patterns mentioned above, angiogenesis mediated by MSCs is critical for the osteogenesis of craniofacial bone. Blood vessels and bone are colocalized within craniofacial tissues and act synergistically in tissue regeneration [28]. MSCs migrate to the defect area and differentiate into osteoblasts to form ossification centers. Functional vasculature provides oxygen and nutrients to the graft microenvironment, facilitates wound healing, improves the integration of the graft into the host tissue, and ensures the long-term survival of the regenerating tissue. Concomitantly, endothelial cells produce osteogenic factors that promote osteogenic differentiation and bone mineralization [29,30]. Therefore, the success of craniofacial regenerative approaches is predicated on successful recruitment, regeneration, or integration of both vascularization and osteogenesis.

Osteogenesis is a lifelong process that regulates bone mass and quality. During this process, Gli1^+^ MSCs proliferate, migrate, and differentiate into osteoblasts according to precisely controlled temporal and spatial patterns. Gli1^+^ MSCs generally do not undergo active division under physiological conditions, and the spatial distribution of these cells can be identified in skeletal tissues. In long bones, Gli1^+^ MSCs reside in the periosteum and metaphysis [31,32]. Craniofacial bones differ from long bones in their developmental origins, osteogenic programs, and structure. Gli1^+^ MSCs are found in all craniofacial sutures, alveolar bone marrow, and neurovascular bundle niches of the teeth [16,33,34]. Understanding the role of Gli1^+^ MSCs in bone development is crucial to comprehend their role in bone tissue repair.

**Figure 1:**
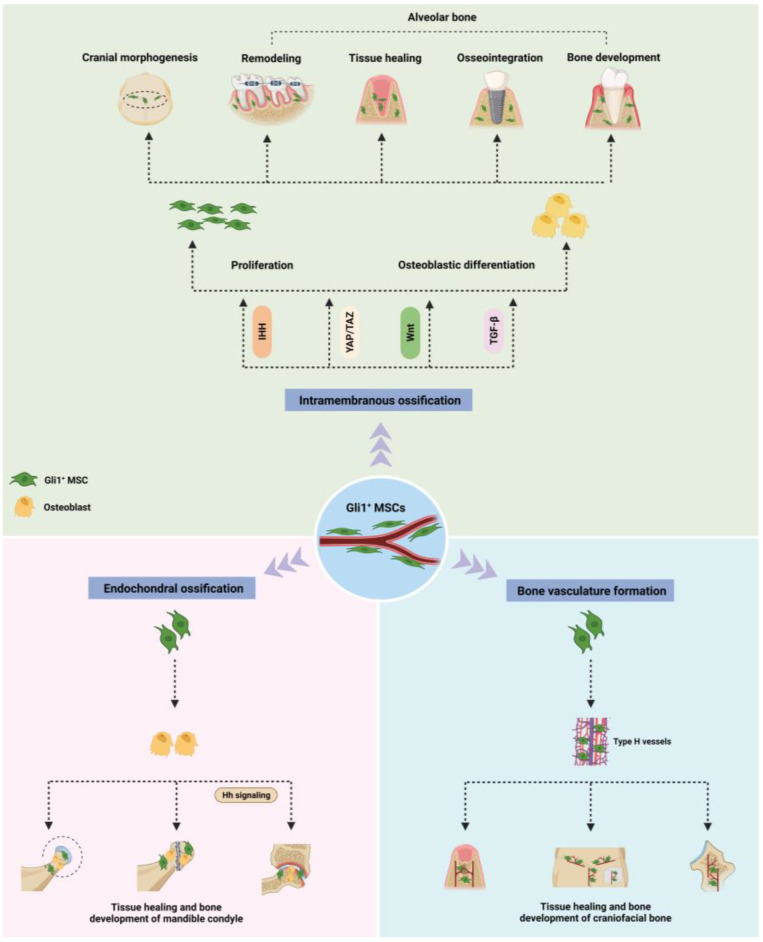
Schematic diagram summarizing the role of Gli1^+^ MSCs in osteogenesis of craniofacial bones. Their role can be outlined in three aspects. First, Gli1^+^ MSCs promote the osteogenesis of skull and alveolar bones formed by intramembrane ossification through enhanced osteoblastic differentiation regulated by multiple signaling pathways. Second, Gli1^+^ MSCs improve tissue repair and bone development of the mandible condyle formed through endochondral ossification by facilitating osteoblastic differentiation. Finally, Gli1^+^ MSCs also display their auxiliary functions in the osteogenesis of craniofacial bone by accelerating the formation of bone vasculature. The image was created using BioRender.com (accessed on 23 August 2023). Abbreviations: Gli1^+^ MSCs, Gli1^+^ mesenchymal stem cells; Hh signaling, Hedgehog (Hh) signaling.

In this review, we comprehensively outlined the pivotal role of Gli1^+^ MSCs in the development and tissue repair of craniofacial bones, and the regulatory mechanisms that govern the osteogenesis of Gli1^+^ MSCs. We summarized the role of Gli1^+^ MSCs in current Gli1-related studies based on the ossification modes of the craniofacial bone: intramembrane ossification, endochondral ossification, and bone vasculature (Figure 1).

## 2. Role of Gli1^+^ MSC in Osteogenesis of Craniofacial Bones Formed through Intramembrane Ossification

Gli1^+^ MSCs exist in multiple tissues and organs, and their functions vary depending on the cell niche environment. Here, we discuss the roles of Gli1^+^ MSCs in the osteogenesis of the skull and alveolar bone. The findings of previous studies on the role of Gli1^+^ MSCs in the osteogenesis of craniofacial bones are summarized in Table 1.

### 2.1. Skull

Skull sutures are fibrous joints that connect the bones of the skull and serve as a dynamic mesenchymal stem cell niche to regulate skull growth and development [48]. Within the sutured stroma, Gli1^+^ cells are the stem cells that support skull remodeling and repair after injury. Lineage tracing experiments, which were utilized to follow the fate of Gli1^+^ MSCs and their progeny in mice, demonstrated that Gli1^+^ MSCs were gradually restricted to the interstitial region of the sutures due to their broad distribution in the periosteum, dura mater, and sutures [16]. Cell quantification and transplantation experiments showed that the function of Gli1^+^ MSCs in the craniofacial bone marrow was much less significant than that of sutured Gli1^+^ MSCs. In addition, ablation of Gli1^+^ MSCs leads to skull growth blockade, osteoporosis, and impaired repair. Similarly, Pietro et al. observed that Gli1^+^ MSCs support craniofacial bone development in the trabecular bones of human skull tissue samples [49]. Targeting Gli1^+^ MSCs is a potential strategy to regulate osteoblast activity and achieve homeostasis in the osteogenic niche.

Normal sutures play a critical role in the three-dimensional growth of the skull. However, premature fusion of cranial sutures can lead to cranial deformities, elevated intracranial pressure, and neurocognitive impairment, which in turn results in decreased quality of life [37]. Facial suture dysfunction can cause maxillary hypoplasia. As a result, there is a strong medical need for effective treatment of cranial suture atresia in clinics. Huang et al. developed a mouse model of rapid palatal suture dilation to investigate the role of Gli1^+^ MSCs in maxillofacial bone remodeling induced by orthopedic forces. They discovered that Gli1^+^ MSCs were rapidly activated during the remodeling process and regulated osteogenesis by altering intracellular Ca^2+^ concentration in response to mechanical stimuli [36]. In one study, skull sutures exhibited greater regenerative ability than other regions of the skull in response to injury. The healing rate of skull defects was also found to be inversely proportional to the distance between the suture and the site of injury [35]. The regenerative potential of sutures makes them promising donor tissues for transplantation to repair skull defects. Yu et al. successfully regenerated functional cranial sutures and restored intracranial pressure, thereby preserving neurocognitive function in mice with craniosynostosis. This was achieved by creating a rectangular defect at the fused coronal suture and implanting Gli1^+^ MSCs from healthy donors along with biodegradable scaffolds [39]. However, further research is required to optimize culture and transplantation methods for Gli1^+^ MSCs using scaffolds.

**Figure 2:**
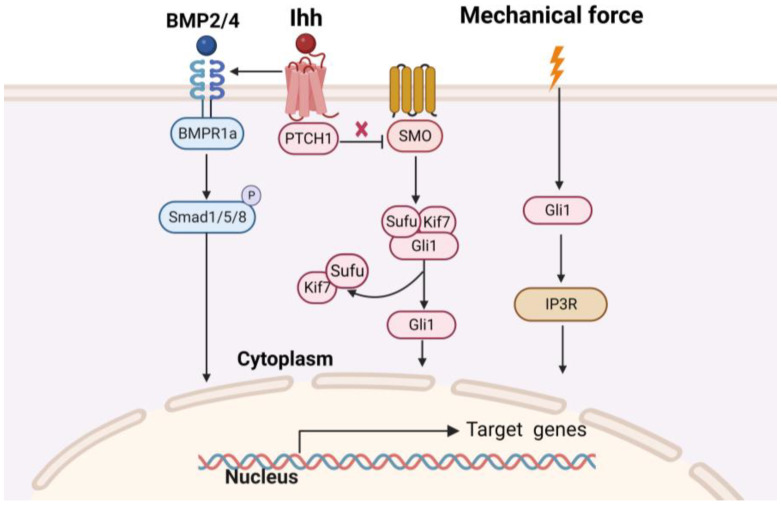
The Schematic diagram to depict the Gli1 signaling pathways that play critical roles in skull morphogenesis[16,36,37,38,50]. The image was created using BioRender.com (accessed on 23 August 2023). Abbreviations: Ihh, India Hedgehog (Ihh); SMO, Smoothened.

The osteogenic fate of Gli1^+^ MSCs is determined by complex signaling pathways that play critical roles in skull morphogenesis (as shown in Figure 2). Any disruption of gene expression patterns or signaling pathways within the niche can disrupt the balance between MSCs proliferation and differentiation in sutures, leading to abnormal skull development. The β-catenin knockout in Gli1^+^ MSCs greatly restricted the activation and osteogenic differentiation abilities of Gli1^+^ MSCs, thereby inhibiting bone remodeling under physiological and dilated conditions [37]. Zhao et al. investigated the effect of Hh signaling on sutured MSCs by generating Gli1-CreERT2; Smo^fl/fl^ mice. They observed a significant reduction in bone volume and severe osteoporosis in the craniofacial bone after eight months of induction, which was reversed by India Hedgehog (Ihh) agonist treatment, which upregulated Gli1 activity [16]. Guo et al. discovered that Gli1^+^ MSCs exhibited active bone morphogenetic protein (BMP) signaling at the skull suture, and conditional inactivation of BMP Receptor 1A led to the downregulation of Hh signaling and suture stenosis. In mice with skull defects, Hh agonists were found to restore morphological and functional disorders resulting from the loss of BMP Receptor 1A and promote skull healing [38]. To gain a better understanding of this cellular regulatory network, additional regulatory factors and mechanisms need to be investigated, establishing a solid foundation for further research on Gli1^+^ MSCs.

### 2.2. Alveolar Bone

The alveolar bone is composed of fascicular bones consisting of thick outer cortical plates and spongy inner cancellous bone. Like other types of bone tissue, alveolar bone undergoes bone remodeling, particularly after tooth eruption, tooth extraction, orthodontic tooth movement (OTM), and masticatory adaptation [51]. In clinical practice, dentists frequently encounter alveolar bone loss caused by various conditions, especially periodontal disease, maxillofacial injury, implantation failure, oral tumors, and subsequent postoperative radiotherapy injury [52]. Therefore, maintenance or regeneration of the alveolar bone is crucial to addressing these issues.

Mounting evidence suggests that endogenous stem cells target periodontal tissues and play a role in regeneration and immune regulation [53]. Men et al. identified Gli1^+^ cells in the adult mouse periodontal ligament as multipotent stem cells capable of differentiating into osteoblasts and contributing to the maintenance of alveolar bone homeostasis via pedigree tracing analysis [41]. Shalehin et al. extracted the maxillary first molars from 8-week-old Gli1-CreERT2/tdTomato mice and transplanted them subcutaneously into wild-type mice. The results demonstrated that Gli1^+^ MSCs gradually expanded from the root surface to the surrounding connective tissue after transplantation, and osterix-positive Gli1^+^ MSCs are found in the regenerated alveolar bone at the root bifurcation [43]. These studies confirm that Gli1^+^ MSCs are an important cell source with high plasticity that promotes periodontal tissue formation and regeneration. However, further research is necessary to explore methods for harnessing the innate regenerative ability of the body and regulating cell homing to improve the application of host stem cells in tissue engineering.

Gli1^+^ MSCs respond to mechanical forces, and mechanical stimulation is a key regulatory factor associated with these cells. Men et al. investigated the effect of physiological bite force on Gli1^+^ MSCs in periodontal tissue by unloading the lower molars relative to one side of the upper molars in adult Gli1-CreERT2; Ai14 mice. The results showed a significant reduction in the number of Gli1^+^ MSCs, alveolar bone height, and relative bone mineral density in the unloaded periodontal tissue compared to the control group [41]. OTM is also dependent on force-induced periodontal cell responses and alveolar bone remodeling. Seki et al. demonstrated the differentiation of Gli1^+^ MSC in the periodontal ligament by placing nickel-titanium coil springs between the upper anterior teeth and first molars. The results showed that after OTM initiation, Gli1^+^ MSCs were amplified on the tension side of the alveolar bone, with Runx2/Gli1 double-positive osteocytes located on the newly formed bone matrix surface [42]. These studies provide functional evidence for the involvement of Gli1^+^ MSCs in alveolar bone remodeling mediated by internal forces.

Implant restoration has become a widely accepted treatment for the restoration of missing teeth owing to its high predictability and success rate, with osseointegration being the key to successful implantation [54,55]. A recent study by Yi et al. identified that Gli1^+^ MSCs and their progeny in alveolar bone play a crucial role in alveolar healing and implant osseointegration [33]. Using three-dimensional imaging based on tissue clearance and pedigree tracing of transgenic mouse models, Yi et al. demonstrated that Gli1^+^ MSCs could be activated and proliferate along blood vessels in the tooth extraction fossa and around the titanium implant. These cells then differentiate into osteocytes and promote osseointegration around the implant. Interestingly, gene ablation of Gli1^+^ MSCs with diphtheria toxin (DTA) resulted in a significant reduction in the bone volume around the implant and failed to form a good bone tissue structure, emphasizing the importance of Gli1^+^ MSCs in successful implant osseointegration.

Understanding the factors that drive Gli1^+^ MSCs differentiation is crucial for the development of novel therapeutic approaches. Xu et al. discovered that knocking out the TGF-β receptor gene in Gli1^+^ MSCs significantly decreased cell proliferation and osteogenic differentiation, leading to a reduction in alveolar bone mass and bone density in mice [40]. Likewise, conditional deletion of β-catenin also hampers the recruitment and osteogenic differentiation of Gli1^+^ MSCs [33]. YAP is a transcription factor that responds to mechanical stimuli and regulates cell behavior by translocating to the nucleus to activate downstream genes [56]. YAP functions as a mechanosensor in Gli1^+^ MSCs, and targeted ablation of the Yap gene in these cells impairs alveolar bone remodeling [44].

## 3. Roles of Gli1^+^ MSCs in Endochondral Ossification of Craniofacial Bones

The mandibular condyle is a crucial site for mandibular growth, and is formed through endochondral ossification. It plays vital roles in mastication and speech [57]. In a previous study, the lineages of Gli1^+^ MSCs in young mice were tracked over a 12-month observation period. The results revealed that the Gli1^+^ MSCs population in the condylar cartilage and soft bone junction of the mandible significantly expanded and spread across almost all trabecular bone surfaces. Gli1^+^ MSCs function as osteoblast progenitor cells and contribute to bone formation and homeostasis of the mandibular condyle [45]. The localization pattern of Gli1^+^ MSCs within the mandibular condyle has been elucidated through lineage tracing experiments conducted in juvenile mice. During the developmental context of the postnatal temporomandibular joint, Gli1^+^ MSCs occupy a niche within the articular cartilage; they might not inherently represent chondroprogenitor cells themselves, thus potentially modulating the activities of adjacent chondroprogenitor counterparts [45,46]. Furthermore, when a condylar fracture was modeled to observe whether Gli1^+^ MSCs could respond to external stimuli during the healing process, researchers found the Gli1^+^ MSCs are locally activated on the fracture sites and migrate to the callus to differentiate into osteoblasts and participate in damaged bone regeneration.

The increasing incidence of temporomandibular joint osteoarthritis (TMJOA) in children and adolescents has raised concerns because of its potential to cause dentomaxillofacial deformities by interfering with condyle growth [58]. Lei et al. discovered that Gli1^+^ MSCs and their progeny could be unevenly distributed and expanded during the development of osteoarthritis, ultimately differentiating into the osteoblast lineage and leading to abnormal subchondral bone remodeling [46]. Inhibition of the Hh signaling pathway in Gli1^+^ MSCs can alleviate subchondral bone pathologies and reduce articular cartilage degeneration. These findings provide in vivo evidence that targeting Hh signaling in Gli1^+^ MSCs can regulate tissue homeostasis in osteoarthritis, offering a potential treatment option for TMJOA.

## 4. Role of Gli1^+^ MSC in the Formation of Bone Vasculature

Bone is a highly vascularized connective tissue. As the main source of oxygen, nutrients, hormones, neurotransmitters, and growth factors delivered to bone cells, the vasculature is indispensable for bone homeostasis and injury repair [29]. Thus, the knowledge and capability to target the molecular mechanisms underlying bone blood vessel function may provide new strategies for treating vasculature-dependent skeletal and systemic diseases that negatively affect bone metabolism. Our previous study showed that the synergistic effect of angiogenesis and the sequential delivery of osteogenic factors could mimic the molecular events of natural bone regeneration [59]. Furthermore, researchers have observed that Gli1^+^ MSCs are concentrated around specific capillaries called type H vessels, which are closely associated with the bone. During bone growth and defect healing, Gli1^+^ MSCs and type H vessels show a close functional correlation, and their expression increases synchronously. Ablation of Gli1^+^ MSCs results in the inhibition of H-type blood vessel formation, which is associated with suppressed bone generation and regeneration [47]. The ability of Gli1^+^ MSCs to regulate angiogenesis and bone remodeling provides a unique advantage in regenerative medicine.

Kusumbe et al. discovered that H-type vessels in specific regions could produce niche signals for osteoprogenitors around the vessels, creating a unique metabolic and molecular microenvironment [29]. Similarly, Yi et al. found that Gli1^+^ MSCs located in the periodontal tissue surround the vascular system, proliferate along blood vessels, and migrate out of the perivascular niche to form new bones after injury [33]. Luo et al. used a three-dimensional imaging technique based on a polyethylene glycol-associated solvent system to examine craniofacial bone development, revealing that Gli1^+^ MSCs at the skull suture were spatially related to osteogenic activity and vascular enrichment [60]. As a subset of perivascular mesenchymal stem cells, Gli1^+^ MSCs play a crucial role in tissue regeneration and repair by modulating the formation of specialized vasculature in adjacent tissues. These findings provide a new theoretical foundation for angiogenesis-targeted bone-tissue engineering.

## 5. Age-Related Distribution in Gli1^+^ MSCs

Due to the plasticity and accessibility of Gli1^+^ MSCs, they emerge as strong contenders in the field of regenerative medicine. With the increasing plausibility of incorporating stem cell-based treatments into medical practice, it is imperative for both researchers and clinicians to acknowledge the inherent diversity within stem cell populations. This diversity holds relevance not only for appraising the efficacy of therapeutic approaches in regenerative medicine, but also for the heterogeneous pool of patients who might contribute autologous or allogeneic stem cells [61,62]. As underscored by the influence of stem cell senescence and malfunction in the natural aging process [63,64,65], the age of donors or patients stands out as a pivotal variable that necessitates careful consideration during both clinical and laboratory assessments of stem cell-centered technologies. Shi and colleagues conducted an assessment of the in vivo temporal and spatial dispersion patterns exhibited by Gli1^+^ MSCs in relation to age. Researchers administered TM to the Gli1-CreERT2; Ai9 mice at 1, 2, 4, or 12 months of age and monitored the Gli1^+^ cells after 24 h. They detected that numerous Gli1^+^ MSCs were distributed among the aggrecan-containing matrix below the growth plate at 1 month of age. By 4 months, however, few Gli1^+^ cells were present under the growth plate, concurrent with the disappearance of the aggrecan-containing matrix from the cancellous bone region. At 12 months of age, essentially no Gli1^+^ MSCs were detectable, even though the growth plate was present. Thus, the Gli1^+^ MSCs beneath the growth plate are abundant in the young postnatal mice, but diminish precipitously with age [17]. Xia et al. also revealed that Gli1^+^ MSCs population residing within the periosteum of long bone are abundant in juvenile mice, but notably diminished by 7 months of age [31].

Future studies should address tissue regeneration in vivo, as this will allow parameters such as aging to be compared using criteria more closely related to the clinical goal of regenerating destroyed or dysfunctional tissues. While the present study offers little in the way of mechanistic explanations for the observed phenomena, it provides analyses of age in an animal model that will be useful for developing future tissue engineering strategies to treat age-related bone defects or degenerative bone diseases.

## 6. The Plasticity of Gli1^+^ MSCs and the Strategies on Navigating Their Differentiation into the Osteogenic Lineage

### 6.1. The Overview of Gli1^+^ MSCs Plasticity

MSCs exhibit noteworthy inter-system and inter-dermal differentiation properties, a phenomenon commonly referred to as the plasticity of MSCs. This plasticity underscores the remarkable capacity of tissue-specific stem cells to undergo a paradigm shift in their developmental trajectory.

MSCs, originating from the mesodermal germ layer, have been the subject of extensive investigation validating their potential to differentiate into diverse mesodermal tissue types. Notably, the introduction of specific cell growth factors to Gli1^+^ MSCs can incite their differentiation into osteoblasts, chondrocytes, and adipocytes [16]. During the regenerative phase following long bone fractures, Gli1^+^ MSCs demonstrate a proclivity for proliferation and migration toward the sites of fracture, subsequently engaging in the differentiation processes that lead to the formation of osteoblasts, osteocytes, and chondrocytes [17]. Similarly, Gli1^+^ MSCs positioned in sutures manifest an ability to align themselves with periosteal cells and osteocytes, thereby contributing substantively to the cyclic renewal and reparative mechanisms governing adult craniofacial bones [16]. Within the oral milieu, the Gli1^+^ MSCs emerges as a pivotal contributor to the generation of periodontal tissues, encompassing the periodontal ligament, cementum, and alveolar bone. These Gli1^+^ MSCs also hold the capacity to serve as progenitor cells for a spectrum of muscular lineages [66]. Evidently, the vascular smooth muscle niche draws from the Gli1^+^ MSC population localized within the neointimal environment of the femoral artery [67]. Furthermore, the diverse capacities of Gli1^+^ MSCs extend to encompass pivotal roles in the cardiac inflow tract and pulmonary apparatus, orchestrating the development of cardiomyocytes, pulmonary vasculature, airway smooth muscle, proximal vascular endothelium, and analogous constituents [13]. Expanding beyond these domains, Gli1^+^ MSCs exhibit a propensity for differentiation along the oligodendrocyte lineage, a characteristic prominently pronounced during the process of myelination. Even in adulthood, these cells partake in the intricate process of remyelination, thereby substantiating their enduring developmental significance [68].

### 6.2. Strategies to Guide the Osteogenic Lineage Differentiation of Gli1^+^ MSCs

#### 6.2.1. The Application of Osteo-Inductive Medium

Various chemical factors, and cytokines/growth factors have been harnessed to effectively steer the osteogenic differentiation trajectory of MSCs [69]. Several chemical compounds have been identified as osteo-inductive agents, capable of inducing MSCs to differentiate into osteoblasts in vitro. Noteworthy among these inducers are 1,25-dihydroxyvitamin D3, L-ascorbic acid, dexamethasone, β-glycerol phosphate, and teriparatide. In the realm of growth factors, a diverse array of agents has been employed in studies focused on bone regeneration. These encompass bone morphogenetic proteins (BMPs), fibroblast growth factors (FGFs), insulin-like growth factors I and II, as well as platelet-derived growth factor, to name a few [70]. Furthermore, the expression of vascular endothelial growth factor (VEGF) within vascularized tissues assumes a pivotal role, orchestrating vascular tissue regeneration within the context of the new bone formation process [71]. The combined utilization of these chemical and cytokine/growth factors represents a potent avenue for modulating and enhancing the osteogenic differentiation of MSCs, thereby bolstering the regenerative prospects within the realm of bone tissue engineering and repair.

#### 6.2.2. Tissue Engineering

Tissue engineering is a multifaceted methodology hinging upon a triad of fundamental components: stem cells, growth factors, and scaffold materials. This approach stands as a firmly established technique, strategically harnessed to modulate the plasticity of Gli1^+^ MSCs toward osteogenic differentiation. Within the realm of tissue engineering, a constellation of cell mobilization factors, including but not limited to granulocyte colony-stimulating factor (G-CSF), stem cell factor (SCF), and stromal cell-derived factor-1 (SDF-1), are adeptly employed to orchestrate the recruitment of endogenous MSCs [72,73]. Concomitantly, an amalgamation of tissue-mimetic materials, growth factors, and regulatory cytokines are judiciously introduced, synergistically fostering an environment conducive to localized microenvironmental optimization, thereby fostering the tissue formation.

The incorporation of extracellular matrix (ECM)-analogous biomaterials within the framework of tissue engineering engenders a bespoke cellular milieu, akin to a nurturing haven and a structural template [74]. These biomaterials assume the role of a cellular “domicile”, offering not only the essential structural components requisite for MSCs support, but also housing pivotal cell signaling motifs and encapsulated growth factors. This orchestration effectively directs cellular anchoring and behavior, expediting the establishment of desired cellular functions [75,76]. The selection of biomaterial scaffolds embraces a diverse array of options, ranging from collagen, hyaluronan, mineralized calcium, and fibrin, to their composite formulations, each manifesting as principal constituents mirroring the architecture of bone ECM. Furthermore, synthetic polymer materials, serving as an exclusive matrix for scaffold fabrication, contribute to the paradigm of matrix scaffolds tailored for the purpose of bone regeneration [77]. Augmentation of these scaffolds with essential inorganic ions, intrinsic to the bone formation process, imparts a propensity to foster the osteogenic differentiation of MSCs, thus amplifying their regenerative potential [78]. Remarkably, the electrostatic charge exhibited at the scaffold surface confers an additional dimension to its functionality. This electrostatic charge exerts a distinctive influence, potentiating the augmentation of osteogenic differentiation within the context of adult stem cells [79]. The collective orchestration of these elements within the realm of biomaterial scaffolds forms an intricate landscape for enhancing the osteogenic differentiation of MSCs, encapsulating both structural support and biochemical guidance, ultimately contributing to the overarching goal of effective tissue engineering for bone repair and regeneration.

Overall, the application administration of Gli1^+^ MSCs in regenerative medicine is promising, and should be the focus of foundational and translational research in the future. Gli1^+^ MSCs-based tissue engineering is expected to offer an idea for reconstructing the craniofacial bone.

## 7. Conclusions

As a promising source of autologous stem cells, Gli1^+^ MSCs have been found to be particularly active in the process of osteogenesis in craniofacial bone following strict lineage-specific differentiation procedures in vivo. The fate of these cells is determined by gene regulatory networks formed by signaling pathways. Further investigation is needed to elucidate the molecular mechanisms that control their regenerative response. This would enhance our understanding of craniofacial bone plasticity and pave the way for the development of novel cell therapies that promote bone regeneration, and ultimately improve patients’ quality of life and reduce healthcare costs.

## Figures and Tables

**Table 1:** Studies of Gli1^+^ MSCs in osteogenesis of craniofacial bone.

Osteogenic Patterns	Anatomical Position	Transgenic Mice Model	Intervention	Related Signaling	Results	References
Intramembranous ossification	Skull	Gli1-LacZ Gli1-CreERT2; tdTomato	Suture and calvarial bone injury	/	1. Gli1^+^ MSCs are the cellular sources for injury repair and bone regeneration 2. The healing rate of calvarial bone is inversely proportional to the distance between the suture and injury site	[35]
		Gli1-LacZG li1-CreERT2; mT/mG	Midpalatal suture expansion	IP_3_R	Gli1^+^ MSCs participated in mechanical force-induced osteogenesis by regulating IP3R-mediated intracellular calcium concentration	[36]
		Gli1-LacZ Gli1-CreERT2; Ai14 Gli1-CreERT2; Ctnnb1^f/f^; Ai14	Calvarial suture expansion	Wnt	1. Gli1^+^ MSCs actively contributes to bone remodeling in response to tensile force 2. Conditional knockout of Ctnnb1 impeded the activation of Gli1^+^ MSCs, subsequently inhibiting the bone restoration under mechanical expansion	[37]
		Gli1-LacZ Gli1-CreERT2; tdTomato Gli1-CreERT2; DTA^f/f^ Gli1-CreERT2; Smoothened^f/f^	Suture and calvarial bone injury	/	1. Gli1^+^ MSCs in the suture mesenchyme give rise to osteogenic fronts, periosteum, and dura 2. Ablation of Gli1^+^ MSCs leads to craniosynostosis, skull growth arrest, and compromised injury repair. 3. Craniofacial bones exhibited severe osteoporosis and reduced bone volume following the blockage of the Hh pathway	[16]
		Gli1-LacZ Gli1-CreERT2; tdTomato Gli1-CreERT2; Bmpr1a^f/f^	Calvarial bone defect; addition of exogenous IHH	IHH/BMP	1. Gli1^+^ MSCs give rise to osteoprogenitors that display active BMP signaling activity within the cranial suture 2. Loss of BMPR1a in Gli1^+^ MSCs disrupts osteoclastogenic activity by decreasing RANKL/OPG ratio and IHH activity 3. Upregulation of Hh signaling helped maintain the balance between osteoclastogenesis and osteogenesis in cranial sutures and partially restored the calvarial bone-healing process in BMPR1a mutant mice	[38]
		Twist1^+/−^ mice Gli1-CreERT2; Twist1^f/f^	Craniosynostosis model; Calvarial bone defect	/	1. The regenerated suture creates a niche into which endogenous Gli1^+^ MSCs migrated, sustaining calvarial bone homeostasis and repair. 2. Using a biodegradable material combined with Gli1^+^ MSCs can successfully regenerate a functional cranial suture in Twist1^+/−^ mice that corrects skull deformity, normalizes intracranial pressure, and rescues neurocognitive behavior deficits.	[39]
	Alveolar bone	Gli1-CreERT2; TGFβR2^f/f^; tdTomato	/	TGF-β	Disrupting TGF-β signaling in Gli1^+^ MSCs leads to a reduction in OSX^+^ alveolar bone cell numbers, disturbance of periodontal homeostasis, and early postnatal alveolar bone loss	[40]
		Gli1-LacZ Gli1-CreERT2; Ai14 Gli1-CreERT2; Ctnnb1^f/f^; Ai14	Physiological occlusal force	Wnt	1. Gli1^+^ MSCs as multipotential stem cells contribute to the periodontium tissue turnover by migrating out of the NVB niche 2. Knockout of β-catenin results in a significant reduction of alveolar bone height and density, and over half of the molar root surface was exposed 3. Extracting one side molar arrested Gli1^+^ MSCs activation in opposing molars, resulting in PDL tissue loss and reduced Wnt activity.	[41]
		Gli1-CreERT2; tdTomato	Orthodontic force	/	Gli1^+^ MSCs in the PDL, as a source of osteoblasts on the tension side of the alveolar bone, can proliferate and differentiate into osteoblasts and fibroblasts during orthodontic tooth movement	[42]
		Gli1-CreERT2; Ai14 Gli1CreERT2; Ai14; eGFP-DTAGli1-creERT2; β-catenin^f/f^; Ai14	Tooth extraction; Implant placement	Wnt	1. Gli1^+^ MSCs were activated and proliferated along blood vessels after tooth extraction, and their progeny contributed to new bone formation 2. The bone volume and density reduction surrounding the implant in the β-catenin ablation group compromised the healing and osseointegration processes.	[33]
		Gli1-CreERT2; tdTomato	Tooth transplantation	/	Gli1^+^ MSCs are localized within the mature PDL exhibited stem cell properties and could differentiate into osteoblasts and osteocytes during alveolar bone regeneration.	[43]
		Gli1-LacZ Gli1-CreERT2; eGFP-DTA Gli1-CreERT2; YAP^f/f^	Orthodontic force	YAP/TAZ	1. MSCs expressing Gli1 can respond to orthodontic force by supplying Runx2^+^ cells for alveolar bone remodeling 2. Conditional ablation of the Yap gene in Gli1^+^ MSCs can suppress osteogenic differentiation and defective bone formation	[44]
Endochondral ossification	Mandibular condyle	Gli1-CreERT2; tdTomato	Condyle fracture and Sham surgery	/	1. Gli1^+^ MSCs are spatially located at the superficial layers of the cartilage and chondro-osseous junction, and contribute to osteoblasts in the subchondral bone during condyle postnatal development. 2. Gli1^+^ MSCs could differentiate into osteoblasts and chondrocytes during condylar fracture healing. 3. Wnt/β-catenin signaling mediates the proliferation and osteogenic differentiation of Gli1^+^ MSCs in vitro.	[45]
		Gli1-CreERT2; tdTomato Gli1-CreERT2;Smofl/fl	TMJOA	Hh	1. Gli1^+^ MSCs are osteogenic progenitors contributing to subchondral bone formation and homeostasis in the mandibular condyle of the temporomandibular joint in vivo. 2. Uneven distribution of osteogenic differentiation of Gli1^+^ MSCs in the subchondral bone leads to abnormal subchondral bone remodeling via Hh signaling activation and to the development of TMJOA. 3. The selective pharmacological inhibition and specific genetic inhibition of Hh signaling in Gli1^+^ MSCs results in improved subchondral bone microstructure, attenuated local immune inflammatory response in the subchondral bone, and reduced degeneration of the articular cartilage.	[46]
Bone vasculature formation.	Vessel	Gli1-LacZ Gli1- creERT2;iDTA	/	/	1. Type H vessels are identified with distinct functional properties to couple angiogenesis to osteogenesis and mediate bone generation. 2. Type H vessels are the preferable vascular subtype in which Gli1^+^ MSCs are adjacently localized, and mediate developmental and regenerative angiogenesis in bone. 3. Gli1^+^ MSC ablation inhibits type H vessel formation associated with suppressed bone generation and regeneration.	[47]

IP_3_R: Inositol 1,4,5-trisphosphate receptor (IP_3_R). Hh: Hedgehog signaling. OSX: Osterix. NVB: Neurovascular bundle. PDL: Periodontal ligament. TMJOA: Temporomandibular joint osteoarthritis. DTA: Diphtheria toxin.

## Data Availability

The data supporting this study’s findings are available from the corresponding author upon reasonable request.
